# Critical review of the current and future challenges associated with advanced *in vitro* systems towards the study of nanoparticle (secondary) genotoxicity

**DOI:** 10.1093/mutage/gew054

**Published:** 2016-11-04

**Authors:** Stephen J. Evans, Martin J. D. Clift, Neenu Singh, Jefferson de Oliveira Mallia, Michael Burgum, John W. Wills, Thomas S. Wilkinson, Gareth J. S. Jenkins, Shareen H. Doak

**Affiliations:** ^1^In Vitro Toxicology Group, Institute of Life Science and Centre for NanoHealth, Swansea Univeristy Medical School, Swansea University, Singleton Park, Swansea SA2 8PP, Wales, UK,; ^2^Faculty of Health Sciences and Life Sciences, School of Allied Health Sciences, De Montfort University, The Gateway, Leicester LE1 9BH, UK,; ^3^Environmental Health Sciences and Research Bureau, Health Canada, 50 Colombine Driveway, Ottawa, Ontario K1A 0K9, Canada and; ^4^Microbiology and Infectious Diseases, Institute of Life Science, MRC CLIMB Centre, Swansea University Medical School, Singleton Park, Swansea SA2 8PP, UK

## Abstract

With the need to understand the potential biological impact of the plethora of nanoparticles (NPs) being manufactured for a wide range of potential human applications, due to their inevitable human exposure, research activities in the field of NP toxicology has grown exponentially over the last decade. Whilst such increased research efforts have elucidated an increasingly significant knowledge base pertaining to the potential human health hazard posed by NPs, understanding regarding the possibility for NPs to elicit genotoxicity is limited. *In vivo* models are unable to adequately discriminate between the specific modes of action associated with the onset of genotoxicity. Additionally, in line with the recent European directives, there is an inherent need to move away from invasive animal testing strategies. Thus, *in vitro* systems are an important tool for expanding our mechanistic insight into NP genotoxicity. Yet uncertainty remains concerning their validity and specificity for this purpose due to the unique challenges presented when correlating NP behaviour *in vitro* and *in vivo*. This review therefore highlights the current state of the art in advanced *in vitro* systems and their specific advantages and disadvantages from a NP genotoxicity testing perspective. Key indicators will be given related to how these systems might be used or improved to enhance understanding of NP genotoxicity.

## Introduction

Due to their unique physical and chemical characteristics, nanoparticles (NPs) exhibit distinctly different properties to their bulk counterparts, which can directly contribute to their alternative biological interaction and subsequent impact (1). Predominantly, physico-chemical characteristics of NPs, including geometry, solubility, surface area, surface reactivity and surface chemistry, have been noted to drive this phenomenon (2). Further, it has been well-documented in recent years that the NP interaction (i.e. biological impact, entry mechanism and intracellular fate) is highly cell type dependent (3).

Concomitant with their production, the commercial application of NPs is constantly increasing. For example, carbon nanotubes (CNTs) have been noted as having a production at the tonne level per year (4) e.g. car tyres, sporting equipment and electronics amongst other applications (5). Thus, understanding the potential adverse impact NPs may pose towards human and environment health is of heightened importance (6). Despite this, the necessary methodologies to enable this approach have been significantly lacking over the years (7,8). Whilst initial efforts within the field were focused towards adapting conventional biochemical testing protocols to overcome NP test system interference issues (9), in recent years, attention has been directed towards alternative testing systems (to *in vivo* strategies), most notably *in vitro* models. Adoption of this perspective was further emphasised through recent legislative change, i.e. the EU cosmetics directive [Directive 86/609/EEC (10) and Directive 2010/63/EU (11)].

Historically, an acute exposure scenario to NPs has been the basis for the plethora of research articles published (12). However, in order to interpret their potential hazard, study of the inevitable human exposure to nanosized materials must be considered accurately. Therefore, emphasis should be directed towards the more realistic repeated, chronic and low-dose exposure to the many different NPs and NP-orientated applications produced (13). With this exposure scenario in mind, the potential for NPs to cause genotoxicity has, most recently, received increased interest and risen to the forefront of nanotoxicology research (7).

Genetic damage can arise either through primary (direct or indirect) or through secondary mechanisms (14). Primary direct DNA damage requires the NPs to locate within the nucleus of a cell, interacting with and leading to physical DNA damage (15). This could result in the formation of DNA lesions and potential mutagenesis due to error-prone repair, physical strand breakages or frameshift mutations (due to the NP size, they could act as an intercalating agent with DNA base pairs) (16). Nonetheless, despite only a few studies showing that NPs can enter the nucleus (17), there is limited evidence supporting the potential for NPs to cause primary direct genotoxicity. Lovrić *et al.* showed quantum dots (diameter 2–3 nm) to be present in the nucleus of MCF-7 human breast cancer cells, yet reported no genotoxicity to occur despite a noted increase in cellular oxidative stress levels after a 15-h exposure at 10 μg/mL. Oxidative stress is considered a key mechanism in primary indirect genotoxicity, occurring from excessive reactive oxygen species (ROS) production which in turn creates an imbalance in the cellular ROS:antioxidant (18,19). To date, research related to deciphering NP genotoxicity has demonstrated (20) or eluded (21) to the notion that NP-associated DNA damage occurs through primary indirect means. In this scenario, the NP is not physically interacting with the DNA, but instead promoting damage through other molecules that either have the capacity to interact with DNA to induce lesions or interfere with DNA replication and cell division (e.g. cell cycle-associated proteins, damage to DNA replication or repair enzymes and/or oxidative stress). Various mechanisms exist by which this could occur, such as a by-product of an induced (pro-)inflammatory response, interaction with cellular components or the high surface reactivity and/or solubility of the NP itself (22,23).

Importantly, both primary mechanisms for DNA damage are solely limited to considering the genotoxic influence of NPs that are associated [i.e. internalised and/or membrane bound (including the extracellular fraction)] with a single cell (type). In this regard, with the NP genotoxicity literature dominated by *in vitro/ex vivo* monoculture analyses, primary indirect particle genotoxicity can arguably be considered the dominant research focus within nanogenotoxicology to date [including the Trojan horse effect (24)]. Yet the main genotoxic mechanism noted *in vivo* is secondary genotoxicity, due to the cell-to-cell interplay that occurs *in vivo* during any foreign body stimulus. It must be stressed therefore, that as most *in vitro* studies in nanogenotoxiciology have not considered this aspect, the true mechanism of NP-associated genotoxicity is not, currently, fully understood.

Secondary genotoxicity is denoted *in vivo* as a result of mechanistic, chronic inflammation caused by activation/recruitment of immune cells, such as macrophages and/or neutrophils (25). These phagocytic cells function *in vivo* as an initial immune defence mechanism against invading microorganisms and aid in the clearance of foreign materials (such as from the lung tissue). Using the lung as an example, as it is the primary route of human exposure to NPs, if clearance of inhaled particulate is unsuccessful it can create a chronic immune cell response. Classically, this would involve a respiratory burst, flooding the tissue with ROS and reactive nitrogen species. This process can result in a vicious circle of free radical production and immune cell recruitment. Indeed, ROS produced via nicotinamide adenine dinucleotide phosphate oxidase activation has been identified as a vital neutrophil recruitment factor in lung tissue (26). Thus, with consideration that NP-associated genotoxicity could also arise through secondary genotoxicity mechanisms, it is vital that such a mechanistic understanding is gained. Not only would this further elucidate the potential hazard posed by NPs to human health, it would provide important information to support the safe-by-design approach of NPs (27,28). Currently however, there are deficiencies in using standard monoculture systems to assess the ability for NPs to instigate secondary genotoxicity. The aim of this critical review, therefore, is to consider the range of currently available advanced *in vitro* systems and their potential to be used to study secondary genotoxicity mechanisms. Focus will be given to the advantages and disadvantages posed by these systems towards deducing NP-associated genotoxicity as well as an outlook on what is needed moving forward to enable such *in vitro* systems to replace *in vivo* testing strategies.

### *In vitro* systems for nano(geno)toxicology research

A recent review by Hartung and Sabbioni highlighted an array of alternative systems that are available for toxicology research (29). A further review highlighted the need for an *in vitro* approach towards nanotoxicology research strategies (predominantly in light of the recent EU directive (Directive 2010/63/EU) (30). Yet there remains a clear void of understanding as to how advanced *in vitro* systems might or could be used for nanogenotoxicology testing. Whilst 2D monocultures have predominantly been used in the field, they exhibit significant flaws that render them irrelevant to be used in testing for NP-associated (secondary) genotoxicity. Nonetheless, as described throughout this critical review, there are numerous advanced, potentially alternative *in vitro* models available that could be more suitable in this specific aspect of the NP toxicology field.

## 2D Monocultures

To date, the majority of *in vitro* studies assessing the potential for NPs to exert a genotoxic response have been performed using 2D monoculture systems. Any 2D *in vitro* system suitable for genotoxicity testing should highly consider cell types recommended by OECD guidelines and which have appropriate genetic stability, low DNA damage background and functional p53 activity (31). In a number of nanogenotoxicology studies however, a contentious issue revolves around the choice of cell type used, because a number of investigations use cell lines that are irrelevant towards the prediction of genetic damage in a target organ (32). Typically, a cell choice will be representative of a region of the body the NP is liable to interact with. For instance, the epithelial layer of the alveolar barrier in the human lung can be (loosely) represented by the A549 cell line or the bronchial region of the human respiratory tract by the bronchial cell lines BEAS-2B or 16HBE14o^−^ (33–39).

Single-cell *in vitro*-based testing techniques, especially those utilising immortalised cell lines, offer the advantages of being relatively low cost, high throughput and reproducible. They are also highly useful when conducting initial cytotoxic screenings and when choosing suitable dose ranges (for *in vivo* studies) (40). However, 2D *in vitro* systems are highly limited with regard to their representation of the *in vivo* tissue. Typically, *in vivo* organ/tissue/cell structures are not the flat planer structures represented by most monocultures, instead they are complex geometrical (e.g. 3D) structures consisting of multiple cell types. Monocultures lack this complexity and structural coordination as well as the specific physiological components, thereby limit inter-, intra- and extracellular communications that influence function, proliferation, differentiation, gene expression profiles and cell death. Therefore, taking these limitations into consideration, current standard *in vitro* genotoxicity tests are associated with a lack of sensitivity and specificity in terms of the cell cultures used. Although 2D monoculture models are largely well-suited to evaluating primary genotoxicity, it is important to note that they are unable to detect mechanisms of secondary genotoxicity. Thus, these disadvantages of 2D *in vitro* assays has resulted in an heightened interest in alternative models to assess NP (secondary) genotoxicology. To date, a number of different, advanced *in vitro* models, as described in Table 1, have been developed varying in their degree of complexity and highlighting the progression of *in vitro* systems potentially available for nanogenotoxicity testing strategies.

**Table 1.** Summary of the advanced *in vitro* systems currently available and used within the field of nano(geno)toxicology

Advanced *in vitro* system	Description	Advantages/disadvantages
Conditioned media treatments	Transfer of conditioned culture media from one cell culture (exposed to e.g. NPs) to another cell culture (i.e. commonly a different cell type).	Straightforward, cost-effective methodology. Considered a crude technique that does not allow cellular interplay. Potential toxicity mediators lost during media transfer.
Co-cultures	Multiple (i.e. two or more) cell types cultured together in the same well which represents a specific organ/tissue type.	Allows for important cellular interplay, and if cultured correctly can represent important cell types (in possible anatomical manner) relevant to (NP) target organ. Relative high cost and laborious nature (requiring expert culture).
Microtissues (e.g. spheroids, organoids)	Cells cultured in a manner that allows them to formulate into an (anatomically correct) geometric structure (e.g. 3D tissue-like structure).	Highly representative of the organ/tissue being studied. Highlights important cellular interplay and anatomical structure. High cost, laborious, short-shelf life and difficult to use for certain biochemical endpoints.
Complex 3D structures	Models that are typically pre-made/purchased and representative of specific organs/tissues.	Closely mimics *in vivo* High cost, laborious, expertise required and not possible to achieve all biochemical endpoints.

The purpose of these advanced models is to create representation of specific organs of the body (e.g. lung, liver, heart and brain), beyond that of monoculture systems (29). Such complex *in vitro* models are being increasingly applied in the field of nanotoxicology, but to date, their use has been limited in assessing the genotoxic potential of NPs, and they likely require some adaptation to support the evaluation of DNA damage endpoints (41).

## The conditioned culture medium approach

Typically, this approach has been applied to investigate the impact of an initial immune response upon other cell types [e.g. barrier cells (epithelial cells)], as it would occur *in vivo*. Although attempting to mimic this scenario, and ongoing biochemical effects, the transfer of conditioned culture medium from one cell type to another following NP treatment is elementary. Arguably the most straightforward means of exploring the impact of extracellular signalling of all available advanced *in vitro* systems, this technique, although considering cellular biochemical interplay, does not reflect the important cell-to-cell interplay that occurs *in vivo*.

An example of this *in vitro* approach is shown by Barlow *et al*., in which type II alveolar epithelial cells were initially treated with carbon black NPs. It was noted that a significant pro-inflammatory response occurred in the epithelial cells, yet when this conditioned cell culture media (containing the pro-inflammatory mediators) was subsequently applied to alveolar macrophages, it proceeded to engage macrophage chemotaxis (42). Further, in the study of Shaw *et al.*, cell culture media was retained from macrophages exposed to diesel exhaust particles (DEPs) and then applied to human umbilical vein endothelial cells (HUVECs). It was observed that the subsequent exposure of the conditioned media promoted a dramatic amplification of monocyte chemotactic protein (MCP-P) and interleukin (IL)-6 in the HUVEC cells (43). An additional example, more recently, showed that conditioned media obtained by treatment of THP-1 macrophages with long CNTs elicited an increased production of IL-1β in Met5a mesothelial cells, which provided important information upon the hazard potential of CNTs with similar physico-chemical characteristics to amosite asbestos fibres (44).

In terms of genotoxicity, the application of conditioned media from one cell type onto the target cells would facilitate a basic, albeit non-specific understanding of the importance of secondary mechanisms for DNA damage. Although, as mentioned above, it would not consider the importance of cellular interplay, and there would be uncertainty associated with the potency of the extracellular mediators exposed to the second cell type, this methodology does eliminate the (many) practical problems associated with using co-cultures to assess secondary genotoxicity, such as the isolation of the different cell types required to ensure analysis is restricted to the target cells in the model. Furthermore, it would enable straightforward use of the already established genotoxic tests, such as the COMET and micronucleus tests, and not require further methodological changes to deduce the potential genotoxicity of any NP type.

## Co-culture systems

The next level of complexity in ‘advanced *in vitro* systems’ involves co-culture models, comprised of either two or more different cell types. Although these systems do not necessarily always form a 3D architecture, they encompass cell-to-cell interplay, which promotes intracellular signalling and molecular cross-talk that closely mimic the *in vivo* environment following inhalation of NPs (such as within an occupational exposure environment). A prime example of such a model is the alveolar airway barrier model developed by Rothen-Rutishauser *et al.* (45). This triple cell co-culture model consists of alveolar lung epithelial cells (commonly A549 cells), as well as human blood monocyte-derived macrophages and dendritic cells constructed in an anatomically associated manner. Since its establishment, the model has been applied to a wide range of studies that have primarily focused on cellular entry mechanisms of NPs and their subsequent cytotoxic, oxidative stress and pro-inflammatory responses. As with any multicellular system, question marks remain as to their superiority beyond monocultures. Recently, this question was investigated with the co-culture model of Rothen-Rutishauser *et al*. (45). It was demonstrated following CNT exposure that the co-culture model showed a greater pro-inflammatory cytokine (measured by tumour necrosis factor-α) and chemokine (measured by IL-8) response compared with the same CNT treatments on monocultures of each independent cell type of the co-culture model (46).

Due to its high relevance in the field, in terms of NP inhalation being the primary route of exposure to the human body, the application of a lung co-culture model has been a recurring theme within the nanotoxicology field. Several research groups have also constructed similar models since 2005, with structural variation (47) as well as differences in the cell types used (48). Similar toxicological questions have been asked of these co-cultures as to the one of Rothen-Rutishauser *et al*. (45), yet limited genotoxicity assessment of any NP type has been conducted using these models. In addition, various other co-culture models have been constructed to represent other organs of the body; e.g. the gut has been a key area of investigation due to the potential ingestion of NPs [e.g. titanium dioxide (TiO_2_) NPs through their application as food additives]. Thus, a co-culture of colon epithelial cells (CaCo-2), THP-1 macrophages and MUTZ-3 dendritic cells have been constructed to assess NP toxicity in healthy and diseased conditions (49). Moreover, a model consisting of CaCo-2 cells and another colon epithelial cell line (HT29-MTX) has also been applied to assess the ability of the gut mucosa to change the physico-chemical characteristics of polystyrene NPs (50).

In all cases, the application of co-culture models to nanotoxicology has primarily been focused on uptake, pro-inflammatory and cytotoxicity studies with a very limited number investigating DNA damage. Yet comparisons have been made between the ability of DEPs (as a bulk sample, not just the NP fraction) to induce DNA damage in both mono- and co-cultured lung epithelial cells and macrophages. It was shown that in a macrophage/lung epithelial co-culture, there was a loss in the ability for DEPs to cause DNA damage when comparing this endpoint (determined by the COMET assay) in mono- and co-culture systems due to the pivotal, ‘protective’ effect that (alveolar) macrophages play on the apical layer of the epithelial layer (51). Moreover, correlation of a model consisting of lung epithelial and endothelial cells with *in vivo* gene expression data following multi-walled CNTs (MWCNTs) exposure indicated, to a small degree, the potential comparability of *in vitro* and *in vivo* models (52). Yet, in this study, the immune response measured was dampened due to the lack of any phagocytic cells present in the *in vitro* system. Nonetheless, the findings of Snyder-Talkington *et al*. are of significant importance in the perspective of alterative *in vivo* model systems. It is also worth highlighting that in their approach, Snyder-Talkington *et al.* used a variety of genes associated with genotoxicity to determine the impact of MWCNTs. Whilst this is not direct genotoxic assessment, it again shows a clear path of how to approach a difficult methodological issue. Whilst it can be considered that the use of co-culture models for genotoxicity assessment would provide significant and useful data on the potential for NPs to cause secondary genotoxicity, they however present with a number of practical issues. The most prominent limitation is the difficulty in isolating each cell type of interest for DNA damage evaluation. An example of this was presented by Jantzen *et al.* who stated that the nucleoids for both epithelial cells and macrophages looked identical under the fluorescence microscopy (51). Thus, the authors were unable to discriminate as to which cell type the scored nucleoids originated from. This issue has also been associated with other genotoxicity testing strategies, such as the γ-H2ax assay. Similarly, via gene-based approaches, the ability to deduce the precise cell type without any initial or real-time identification of the different cell types in the multicellular system is a significant disadvantage in using these advanced models. In an attempt to overcome this issue however, Clift *et al.* who have devised a straightforward cell phenotypic flow cytometry-based approach to determine each specific cell type of a co-culture system following their isolation (M. J. D. Clift *et al.*, unpublished). Yet, irrespective of this approach, due to the specific methodological protocols associated with some individual genotoxicity tests, it will not always be possible to identify the different cell types (e.g. the COMET assay, where all cells are lysed prior to analysis). For assays that involve microscopy-based techniques, cell identification is potentially less problematic and could therefore be applied to tests such as the cytokinesis block micronucleus (CBMN) assay, as well as the flow cytometric version of this assay (53), where cells without the correct fluorescent label could easily be excluded from scoring. Irrespective of this potential solution, there remains a significant issue with most, if not all, currently validated *in vitro* mammalian genotoxicity tests where two or more cell types are cultured together.

## Microtissues (spheriods/organoids)

Moving further into *in vitro* complexity, microtissues show an additional level of *in vivo* association to multicellular systems. A major disadvantage of co-culture models is that they lack a specific 3D architecture, a significant characteristic of *in vivo* tissue. Alternatively, 3D cellular microtissues, where cells are cultured in a manner that supports their growth in a 3D tissue-like structure, replicate *in vivo* tissue behaviour (54). It must be noted though that microtissues can be also be referred to as spheroids and/or organoids. Various approaches have been developed in forming these 3D cell aggregates and include the use of bioreactors and microfluidic devices, as well as formation of hanging drop cultures, together with layered and matrix scaffolds (54,55).

Due to its versatility to integrate with other assays, ease of use and scalability, as well as the ability to produce homogenous sized spheroid systems, the hanging drop method is of common interest throughout the *in vitro* community. This technique can be applied to a number of different cell types and is considered an ideal 3D model for nanotoxicology strategies as their structure provides a biological barrier in addition to augmented *in vivo*-like cell functionality. Thus, these *in vitro* systems can be used to mimic any of the body’s organ type structure and therefore represent exposure via any of the key routes to the human body (e.g. inhalation, injection, ingestion and skin exposure) of NPs and NP-associated applications. Indeed, comparison between monocultures and spheroids treated with both gold NPs and quantum dots demonstrated that lower toxicity occurred in spheroids compared with the 2D monocultures (56). Reason for this is that the layered cell 3D structure offered by these models mean that particle size and agglomeration becomes an even greater consideration as it greatly impacts on penetration and localisation (Huang *et al.*, 2012).

One key organ of interest, other than that of the lung, in nanotoxicology is the liver, as toxicokinetics studies to date have demonstrated that it is often the primary site of accumulation following access of NPs that gain access into the systemic circulation (57,58). *In vivo* liver damage associated with nanomaterial toxicity has already been reported (59–61). Commercial Insphero™ liver microtissues have been utilised in the genotoxic assessment of a panel of NPs including zinc oxide (ZnO), MWCNTs and TiO_2_ NPs. Each NP type induced DNA damage at varying treatment doses as measured by the COMET assay, with subsequent repeated exposure increasing DNA damage levels (62).

**Figure 1.**
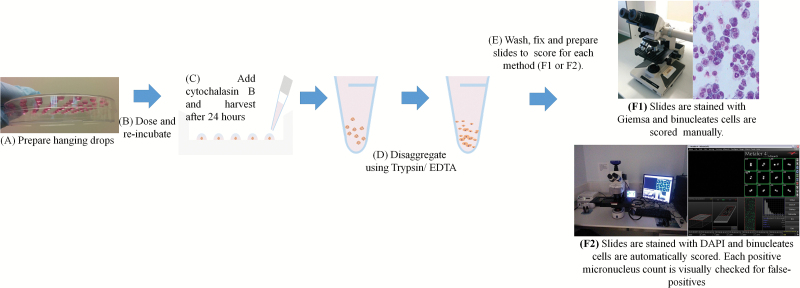
Scheme outlining the procedure associated with determining the micronucleus frequencies in spheroid cultures (HepG2 hanging drop cultures) exposed to dextran-coated Fe_2_O_3_ and Fe_3_O_4_ NPs.

Genotoxicity studies with spheroids to date have only evaluated DNA damage induced by NPs with the COMET assay. Thus, the COMET assay is typically overly sensitive and is also not able to detect aneugenicity; the regulatory *in vitro* testing battery therefore does not include the COMET assay and is restricted to mutation tests that quantify different forms of fixed genetic damage including the chromosome aberration assay, micronucleus assay or point mutation analysis. However, application of these latter test systems to any microtissue model is not without its difficulties that require significant attention in order to deduce the full advantages posed by these systems for genotoxicity testing strategies. Nonetheless, there are exciting developments in the field with ongoing research to optimise the application of the micronucleus assay with HepG2 liver spheroids, allowing the extension of this test to take advantage of the benefits offered by improved tissue structure in microtissues (Figure 1) and to apply it towards exploring NP-associated (secondary) genotoxicity (63).

## 3D Reconstructed tissue models

Finally, beyond the previously discussed advanced *in vitro* models that can be adapted to meet the needs of the different human exposure routes to NPs (and their applications), a specific form of *in vitro* system can be used as regards NP skin exposures. These systems, known as 3D reconstructed skin models, have received increased attention in the genotoxicity field but as yet only limited investigation for NP-associated genotoxicity. A task force was initiated by the European commission led by the European Centre for the Validation of Alternative Methods (ECVAM) to develop *in vitro* skin model-based assays (64). These systems consider the important cellular interplay that co-culture models present, however with the specific geometry and relevance that microtissues exhibit. However, further to these combined advantages of other *in vitro* model systems, 3D tissue structures further exhibit *in vivo* characteristics, e.g. connective tissue, which extends further the *in vivo* relevance of these *in vitro* systems.

This spawned a variety of 3D-reconstructed skin culture models such as the EpiDerm™, SenCeeTox™, KeraSkin™ and Straticell that represent a first point of contact following exposure of a cosmetic product and for skin sensitisation assays. These models are designed to represent a considered ‘normal’ epidermis with discernible basal, spinous and granular layers covered with a developed stratum corneum. Additionally, they are comprised of human keratinocytes, removing the requirement for the rat liver S9 preparation as some models, such as the EpiDerm™ have xenobiotic metabolism relevant to human skin (65). The EpiDerm™ tissue has been applied to a limited number of nanotoxicology studies including the assessment of skin irritation by cerium oxide and silicon dioxide NPs (66,67). KeraSkin™ has also been compared with an *in vivo* model following treatment with ZnO NPs where skin erosion and irritation were assessed by changes in cell viability. This study demonstrated that the *in vitro* data were comparable to the *in vivo* response with the reconstructed skin model (68).

**Figure 2.**
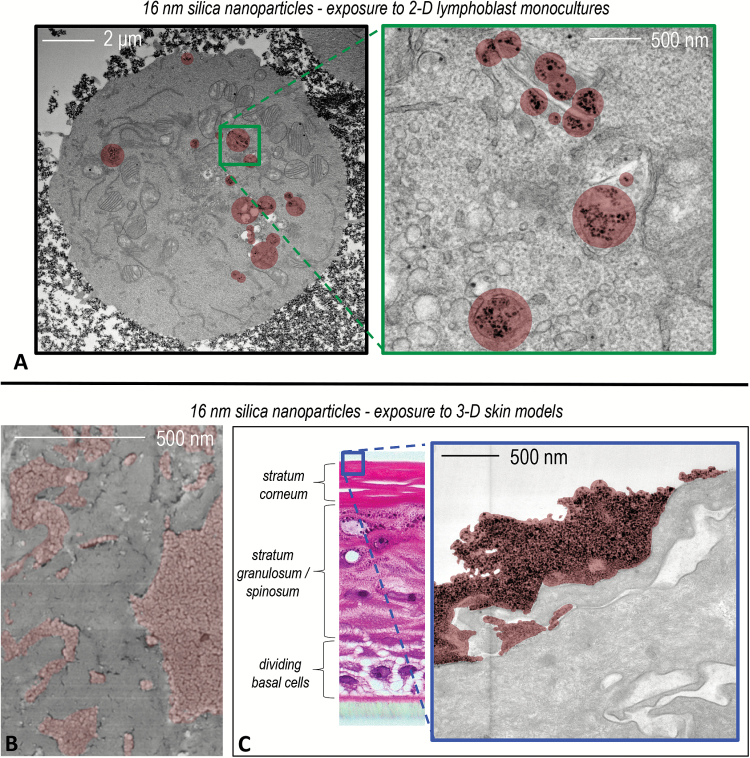
Comparison of 16 nm silica nanoparticle (NP) exposures in traditional 2D monocultures versus the 3D reconstructed skin model: 16 nm silica NP exposure to 2D monoculture cells resulted in cell membrane binding, uptake and consequentially concentration-dependent (geno)toxicity (**A**). Cryogenic scanning electron microscopy permitted the deposition state and surface coverage of the NPs (coloured red) to be assessed after topical inoculation onto the stratum corneum barrier layer of the 3D tissue model (**B**). Re-imaging after 72 h exposure in transverse section showed that the stratum corneum layer was an effective barrier to the silica NPs (red), as they only penetrated the very outermost layers of the stratum corneum layer (**C**; relative position of the electron micrograph in context of the complete model cross section shown by haematoxylin and eosin micrograph inset left). Consequently, no (geno)toxicity was found regardless of concentration, as the 3D microarchitecture of the model prevented silica NP exposure to the living cells of the tissue.

The application of models such as these for the assessment of NP genetic damage is in contrast limited. Despite this, there has been an extensive international effort to adapt the CBMN assay for the use with the EpiDerm™ model, now known as the 3D reconstructed skin micronucleus assay (RSMN). The RSMN assay has been undergoing pre-validation for the last 8 years with a standardised protocol in place and very good reproducibility (69–71). Efforts are now being placed on increasing throughput of the RSMN assay by the use of automated techniques such as the Metasystems Slide Scanning Platform (Metafer) (72). Although the RSMN assay is well accepted and offers a model of true barrier representation, which has been applied to evaluate chemicals, its application in nano(geno)toxicology remains limited. To progress this, recent research has focused on comparing NP exposure, uptake and genotoxic response between 2D monocultures and the EpiDerm™ model. This research utilised cryogenic scanning electron microscopy to characterise NP dermal deposition and cell uptake assessment by transmission electron microscopy comparing the 2D and 3D models (Figure 2). The data gathered demonstrated the importance of 3D model architecture as the EpiDerm™ stratum coreum was an effective barrier to subcutaneous penetration and consequently preventing genotoxicity in the dividing basal cells of the model. In comparison, 2D results demonstrated NP uptake and dose-dependant genotoxicity (Figure 2) (73).

## Summary and outlook

With growing pressure to move away from animal-based testing, there are constant advances within the field of *in vitro* culture technologies and models. Novel approaches involving manipulations of the current state of the art are developing at a fast pace, resulting in a range of *in vitro* models and systems that represent a wide variety of human organs and tissues, each of which are progressing significantly towards mimicking the complete *in vivo* scenario and subsequent response. It is important to note however that there remains a void in understanding how truly alike the currently available *in vitro* systems are to the *in vivo* scenario. Whilst some of the literature eluded to in the present manuscript (52, 69) suggests that currently used *in vitro* systems exhibit similarities to *in vivo*, it is abundantly clear within the field that more efforts must be given towards actually comparing and confirming the proximity of such models to *in vivo*. Whilst this would begin to achieve the visions of the 3Rs in nano(geno)toxicology research (30), emphasis must also be towards enabling *in vitro* systems to mimic the human body and not just a replacement for animal experimentation.

In the current environment however, the options of advanced *in vitro* models represent a reasonable choice for specific organ/tissue analysis, as well as a range of biochemical testing strategies. However, unfortunately it is not a simple case of selecting such a model and applying them towards standardised hazard assessment testing strategies. Consequently, for genotoxicity testing, there remain a number of limitations and issues yet to be solved to truly deliver an *in vitro* system that is adequate for use. Furthermore, the complications of working with NPs raise additional technical hurdles that need to be overcome to allow the inclusion of advanced *in vitro* models in nanogenotoxicology testing. Nonetheless, these alternative models offer significant promise and advantage over the current, gold standard 2D monoculture systems including:

1. The ability to employ more realistic repeated or chronic exposures due to the enhanced long-term stability of the more complex multicellular architecture.
2. Multicellular models provide greater representation of *in vivo* organs and tissues, thereby more closely mimicking the cell-to-cell interplay that could influence behaviour following exposure to exogenous agents.
3. Availability of multiple tissue/organ systems to study intertissue/organ translocation, accumulation and (geno)toxicity profiles.
4. The ability to evaluate a wider spectrum of modes of action, particularly the potential induction of secondary genotoxicity.

Despite the current flaws and limitations, the research area of advanced *in vitro* systems is an exciting field offering substantial advantages to aid the reduction of animal testing and promote the 3Rs principles in the genotoxicity testing of NPs.

## Funding

This work was supported by the Engineering and Physical Sciences Research Council (EP/K502935/1).

Conflict of interest statement: None declared.

## References

[1] Oberdörster G. Stone V. and Donaldson K (2007) Toxicology of nanoparticles: a historical perspective. Nanotoxicology, 1, 2–25.

[2] Bouwmeester H. Brandhoff P. Marvin H. J. P. Weigel S. and Peters R. J. B (2014) State of the safety assessment and current use of nanomaterials in food and food production. Trends Food Sci. Technol., 40, 200–210.

[3] Nazarenus M. Zhang Q. Soliman M. G. (2014) In vitro interaction of colloidal nanoparticles with mammalian cells: what have we learned thus far? Beilstein J. Nanotechnol., 5, 1477–1490. 25247131 10.3762/bjnano.5.161 PMC4168913

[4] Fadel T (2015) Realizing the Promise of Carbon Nanotubes: Challenges, Opportunities, and the Pathway to Commercialization http://www.nano.gov/sites/default/files/pub_resource/2014_nni_cnt_tech_meeting_report.pdf (accessed June 13, 2016).

[5] De Volder M. F. Tawfick S. H. Baughman R. H. and Hart A. J (2013) Carbon nanotubes: present and future commercial applications. Science, 339, 535–539. 23372006 10.1126/science.1222453

[6] Donaldson K. Brown D. Clouter A. Duffin R. MacNee W. Renwick L. Tran L. and Stone V (2002) The pulmonary toxicology of ultrafine particles. J. Aerosol Med., 15, 213–220. 12184871 10.1089/089426802320282338

[7] Singh N. Manshian B. Jenkins G. J. Griffiths S. M. Williams P. M. Maffeis T. G. Wright C. J. and Doak S. H (2009) NanoGenotoxicology: the DNA damaging potential of engineered nanomaterials. Biomaterials, 30, 3891–3914. 19427031 10.1016/j.biomaterials.2009.04.009

[8] Arora S. Rajwade J. M. and Paknikar K. M (2012) Nanotoxicology and in vitro studies: The need of the hour. Toxicol. Appl. Pharmacol., 258, 151–165. 22178382 10.1016/j.taap.2011.11.010

[9] Oostingh G. J. Casals E. Italiani P. (2011) Problems and challenges in the development and validation of human cell-based assays to determine nanoparticle-induced immunomodulatory effects. Part. Fibre Toxicol., 8, 1–21. 21306632 10.1186/1743-8977-8-8 PMC3045340

[10] EC (1986) Council Directive 86/609/EEC of 24 November 1986 on the approximation of laws, regulations and administrative provisions of the Member States regarding the protection of animals used for experimental and other scientific purposes. J Eur. Union, L358, 1–59. 20397315

[11] EC (2010) Directive 2010/63/EU of the European Parliament and of the Council of 22 September 2010 on the protection of animals used for scientific purposes J Eur. Union, 28, 82–128.

[12] Stone V. Miller M. R. Clift M. J. D. (2016) Nanomaterials vs ambient ultrafine particles: an opportunity to exchange toxicology knowledge. Environ. Health Perspect. In press. 10.1289/EHP424 PMC5933410 29017987

[13] Arts J. H. E. Hadi M. Keene A. M. (2014) A critical appraisal of existing concepts for the grouping of nanomaterials. Regul. Toxicol. Pharmacol., 70, 492–506. 25108058 10.1016/j.yrtph.2014.07.025

[14] Doak S. H. Liu Y. and Chen C (2012) Chapter 14—genotoxicity and cancer A2—Fadeel, Bengt. In Pietroiusti A. and Shvedova A. A (eds), Adverse Effects of Engineered Nanomaterials. Academic Press, Boston, MA, pp. 243–261.

[15] Schins R. P. and Knaapen A. M (2007) Genotoxicity of poorly soluble particles. Inhal. Toxicol., 1, 189–198. 10.1080/08958370701496202 17886067

[16] Magdolenova Z. Collins A. Kumar A. Dhawan A. Stone V. and Dusinska M (2014) Mechanisms of genotoxicity. A review of in vitro and in vivo studies with engineered nanoparticles. Nanotoxicology, 8, 233–278. 23379603 10.3109/17435390.2013.773464

[17] Lovrić J. Cho S. J. Winnik F. M. and Maysinger D (2005) Unmodified cadmium telluride quantum dots induce reactive oxygen species formation leading to multiple organelle damage and cell death. Chem. Biol., 12, 1227–1234. 16298302 10.1016/j.chembiol.2005.09.008

[18] Donaldson K. Poland C. A. and Schins R. P (2010) Possible genotoxic mechanisms of nanoparticles: criteria for improved test strategies. Nanotoxicology, 4, 414–420. 20925449 10.3109/17435390.2010.482751

[19] Stone V. and Donaldson K (2006) Nanotoxicology: signs of stress. Nat. Nano., 1, 23–24. 10.1038/nnano.2006.69 18654137

[20] Singh N. Jenkins G. J. S. Nelson B. C. Marquis B. J. Maffeis T. G. G. Brown A. P. Williams P. M. Wright C. J. and Doak S. H (2012) The role of iron redox state in the genotoxicity of ultrafine superparamagnetic iron oxide nanoparticles. Biomaterials, 33, 163–170. 22027595 10.1016/j.biomaterials.2011.09.087

[21] Ghosh M. Sinha S. Chakraborty A. Mallick S.K. Bandyopadhyay M. and Mukherjee A (2012) In vitro and in vivo genotoxicity of silver nanoparticles. Mutat. Res., 749, 60–69. 22960309 10.1016/j.mrgentox.2012.08.007

[22] Duffin R. Tran L. Brown D. Stone V. and Donaldson K (2007) Proinflammogenic effects of low-toxicity and metal nanoparticles in vivo and in vitro: highlighting the role of particle surface area and surface reactivity. Inhal. Toxicol., 19, 849–856. 17687716 10.1080/08958370701479323

[23] Monteiller C. Tran L. MacNee W. Faux S. Jones A. Miller B. and Donaldson K (2007) The pro-inflammatory effects of low-toxicity low-solubility particles, nanoparticles and fine particles, on epithelial cells in vitro: the role of surface area. Occup. Environ. Med., 64, 609–615. 17409182 10.1136/oem.2005.024802 PMC2092561

[24] Hsiao I. L. Hsieh Y. K. Wang C. F. Chen I. C. and Huang Y. J (2015) Trojan-horse mechanism in the cellular uptake of silver nanoparticles verified by direct intra- and extracellular silver speciation analysis. Environ. Sci. Technol., 49, 3813–3821. 25692749 10.1021/es504705p

[25] Bartek J. Mistrik M. and Bartkova J (2010) Long-distance inflammatory and genotoxic impact of cancer in vivo. Proc. Natl Acad. Sci. USA, 107, 17861–17862. 20926747 10.1073/pnas.1013093107 PMC2964237

[26] Marriott H. M. Jackson L. E. Wilkinson T. S. (2008) Reactive oxygen species regulate neutrophil recruitment and survival in pneumococcal pneumonia. Am. J. Resp. Crit. Care Med., 177, 887–895. 18202350 10.1164/rccm.200707-990OC PMC2643216

[27] Ai J. Biazar E. Jafarpour M. Montazeri M. Majdi A. Aminifard S. Zafari M. Akbari H. R. and Rad H. G (2011) Nanotoxicology and nanoparticle safety in biomedical designs. Int. J. Nanomed., 6, 1117–1127. 10.2147/IJN.S16603 PMC3118686 21698080

[28] Arts J. H. E. Hadi M. Irfan M.-A. (2015) A decision-making framework for the grouping and testing of nanomaterials (DF4nanoGrouping). Regul. Toxicol. Pharmacol., 71, S1–S27. 25818068 10.1016/j.yrtph.2015.03.007

[29] Hartung T. and Sabbioni E (2011) Alternative in vitro assays in nanomaterial toxicology. Nanomed. Nanobiotechnol., 3, 545–573. 10.1002/wnan.153 21766468

[30] Burden N. Aschberger K. Chaudhry Q. (2016) Aligning nanotoxicology with the 3Rs: what is needed to realise the short, medium and long-term opportunities? Nano Today. 2016 July [Epub ahead of print], doi:10.1016/j.nantod.2016.06.007 10.1016/j.yrtph.2017.10.021 29069581

[31] Doak S. H. Manshian B. Jenkins G. J. S. and Singh N (2012) In vitro genotoxicity testing strategy for nanomaterials and the adaptation of current OECD guidelines. Mutat. Res., 745, 104–111. 21971291 10.1016/j.mrgentox.2011.09.013 PMC4028084

[32] Rothen-Rutishauser B. Blank F. Muhlfeld C. and Gehr P (2008) In vitro models of the human epithelial airway barrier to study the toxic potential of particulate matter. Expert Opin. Drug Metab. Toxicol., 4, 1075–1089. 18680442 10.1517/17425255.4.8.1075

[33] Wang Y. Cui H. Zhou J. Li F. Wang J. Chen M. and Liu Q (2015) Cytotoxicity, DNA damage, and apoptosis induced by titanium dioxide nanoparticles in human non-small cell lung cancer A549 cells. Environ. Sci. Pollut. Res., 22, 5519–5530. 10.1007/s11356-014-3717-7 25339530

[34] Foldbjerg R. Dang D. A. and Autrup H (2011) Cytotoxicity and genotoxicity of silver nanoparticles in the human lung cancer cell line, A549. Arch. Toxicol., 85, 743–750. 20428844 10.1007/s00204-010-0545-5

[35] Di Bucchianico S. Migliore L. Marsili P. Vergari C. Giammanco F. and Giorgetti E (2015) Cyto- and genotoxicity assessment of Gold nanoparticles obtained by laser ablation in A549 lung adenocarcinoma cells. J. Nanopart. Res., 17, 1–14.

[36] Kim H. R. Kim M. J. Lee S. Y. Oh S. M. and Chung K. H (2011) Genotoxic effects of silver nanoparticles stimulated by oxidative stress in human normal bronchial epithelial (BEAS-2B) cells. Mutat. Res., 726, 129–135. 21945414 10.1016/j.mrgentox.2011.08.008

[37] Nymark P. Catalán J. Suhonen S. 2013 Genotoxicity of polyvinylpyrrolidone-coated silver nanoparticles in BEAS 2B cells. Toxicology, 313, 38–48. 23142790 10.1016/j.tox.2012.09.014

[38] Cozens A. L. Yezzi M. J. Kunzelmann K. Ohrui T. Chin L. Eng K. Finkbeiner W. E. Widdicombe J. H. and Gruenert D. C (1994) CFTR expression and chloride secretion in polarized immortal human bronchial epithelial cells. Am. J. Respir. Cell Mol. Biol., 10, 38–47. 7507342 10.1165/ajrcmb.10.1.7507342

[39] Manshian B. B. Jenkins G. J. Williams P. M. (2013) Single-walled carbon nanotubes: differential genotoxic potential associated with physico-chemical properties. Nanotoxicology, 7, 144–156. 22263934 10.3109/17435390.2011.647928

[40] Clift M. J. Endes C. Vanhecke D. Wick P. Gehr P. Schins R. P. Petri-Fink A. and Rothen-Rutishauser B (2014) A comparative study of different in vitro lung cell culture systems to assess the most beneficial tool for screening the potential adverse effects of carbon nanotubes. Toxicol. Sci., 137, 55–64. 24284789 10.1093/toxsci/kft216

[41] Clift M. J. Gehr P. and Rothen-Rutishauser B (2011) Nanotoxicology: a perspective and discussion of whether or not in vitro testing is a valid alternative. Arch. Toxicol., 85, 723–731. 20499226 10.1007/s00204-010-0560-6

[42] Barlow P. G. Clouter-Baker A. Donaldson K. MacCallum J. and Stone V (2005) Carbon black nanoparticles induce type II epithelial cells to release chemotaxins for alveolar macrophages. Part. Fibre Toxicol., 2, 11. 16332254 10.1186/1743-8977-2-11 PMC1325251

[43] Shaw C. A. Robertson S. Miller M. R. Duffin R. Tabor C. M. Donaldson K. Newby D. E. and Hadoke P. W. F (2011) Diesel exhaust particulate–exposed macrophages cause marked endothelial cell activation. Am. J. Resp. Cell Mol. Biol., 44, 840–851. 10.1165/rcmb.2010-0011OC 20693402

[44] Murphy F. Schinwald A. Poland C. and Donaldson K (2012) The mechanism of pleural inflammation by long carbon nanotubes: interaction of long fibres with macrophages stimulates them to amplify pro-inflammatory responses in mesothelial cells. Part. Fibre Toxicol., 9, 8. 22472194 10.1186/1743-8977-9-8 PMC3352110

[45] Rothen-Rutishauser B. M. Kiama S. G. and Gehr P (2005) A three-dimensional cellular model of the human respiratory tract to study the interaction with particles. Am. J. Resp. Cell Mol. Biol., 32, 281–289. 10.1165/rcmb.2004-0187OC 15640437

[46] Müller L. Riediker M. Wick P. Mohr M. Gehr P. and Rothen-Rutishauser B (2009) Oxidative stress and inflammation response after nanoparticle exposure: differences between human lung cell monocultures and an advanced three-dimensional model of the human epithelial airways. J. R. Soc. Interface, 7, S27–S40. 19586954 10.1098/rsif.2009.0161.focus PMC2843981

[47] Snyder-Talkington B. N. Schwegler-Berry D. Castranova V. Qian Y. and Guo N. L (2013) Multi-walled carbon nanotubes induce human microvascular endothelial cellular effects in an alveolar-capillary co-culture with small airway epithelial cells. Part. Fibre Toxicol., 10, 1–14. 23903001 10.1186/1743-8977-10-35 PMC3750368

[48] Alfaro-Moreno E. Nawrot T. S. Vanaudenaerde B. M. Hoylaerts M. F. Vanoirbeek J. A. Nemery B. and Hoet P. H (2008) Co-cultures of multiple cell types mimic pulmonary cell communication in response to urban PM10. Eur. Respir. J., 32, 1184–1194. 18653652 10.1183/09031936.00044008

[49] Susewind J. de Souza Carvalho-Wodarz C. Repnik U. Collnot E.-M. Schneider-Daum N. Griffiths G. W. and Lehr C. -M (2016) A 3D co-culture of three human cell lines to model the inflamed intestinal mucosa for safety testing of nanomaterials. Nanotoxicology, 10, 53–62. 25738417 10.3109/17435390.2015.1008065

[50] Walczak A. P. Kramer E. Hendriksen P. J. M. Helsdingen R. van der Zande M. Rietjens I. M. C. M. and Bouwmeester H (2015) In vitro gastrointestinal digestion increases the translocation of polystyrene nanoparticles in an in vitro intestinal co-culture model. Nanotoxicology, 9, 886–894. 25672814 10.3109/17435390.2014.988664

[51] Jantzen K. Roursgaard M. Desler C. Loft S. Rasmussen L. J. and Møller P (2012) Oxidative damage to DNA by diesel exhaust particle exposure in co-cultures of human lung epithelial cells and macrophages. Mutagenesis, 27, 693–701. 22869610 10.1093/mutage/ges035

[52] Snyder-Talkington B. N. Dong C. Zhao X. Dymacek J. Porter D. W. Wolfarth M. G. Castranova V. Qian Y. and Guo N. L (2015) Multi-walled carbon nanotube-induced gene expression in vitro: concordance with in vivo studies. Toxicology, 328, 66–74. 25511174 10.1016/j.tox.2014.12.012 PMC4308557

[53] Bryce S. M. Shi J. Nicolette J. Diehl M. Sonders P. Avlasevich S. Raja S. Bemis J. C. and Dertinger S. D (2010) High content flow cytometric micronucleus scoring method is applicable to attachment cell lines. Environ. Mol. Mutagen., 51, 260–266. 19950402 10.1002/em.20544 PMC2841230

[54] Mehta G. Hsiao A. Y. Ingram M. Luker G. D. and Takayama S (2012) Opportunities and challenges for use of tumor spheroids as models to test drug delivery and efficacy. J. Control. Release, 164, 192–204. 22613880 10.1016/j.jconrel.2012.04.045 PMC3436947

[55] Breslin S. and O’Driscoll L (2013) Three-dimensional cell culture: the missing link in drug discovery. Drug Discov. Today, 18, 240–249. 23073387 10.1016/j.drudis.2012.10.003

[56] Lee J. Lilly G. D. Doty R. C. Podsiadlo P. and Kotov N. A (2009) In vitro toxicity testing of nanoparticles in 3D cell culture. Small, 5, 1213–1221. 19263430 10.1002/smll.200801788

[57] Wang B. He X. Zhang Z. Zhao Y. and Feng W (2013) Metabolism of nanomaterials in vivo: blood circulation and organ clearance. Acc. Chem. Res., 46, 761–769. 23964655 10.1021/ar2003336

[58] Roy R. Kumar S. Tripathi A. Das M. and Dwivedi P. D (2014) Interactive threats of nanoparticles to the biological system. Immunol. Lett., 158, 79–87. 24316409 10.1016/j.imlet.2013.11.019

[59] Choi J. E. Kim S. Ahn J. H. Youn P. Kang J. S. Park K. Yi J. and Ryu D.Y (2010) Induction of oxidative stress and apoptosis by silver nanoparticles in the liver of adult zebrafish. Aquat. Toxicol., 100, 151–159. 20060176 10.1016/j.aquatox.2009.12.012

[60] Park E. J. Bae E. Yi J. Kim Y. Choi K. Lee S. H. Yoon J. Lee B. C. and Park K (2010) Repeated-dose toxicity and inflammatory responses in mice by oral administration of silver nanoparticles. Environ. Toxicol. Pharmacol., 30, 162–168. 21787647 10.1016/j.etap.2010.05.004

[61] Sharma V. Singh P. Pandey A. K. and Dhawan A (2012) Induction of oxidative stress, DNA damage and apoptosis in mouse liver after sub-acute oral exposure to zinc oxide nanoparticles. Mutat. Res., 745, 84–91. 22198329 10.1016/j.mrgentox.2011.12.009

[62] Kermanizadeh A. Gaiser B. K. Johnston H. Brown D. M. and Stone V (2014) Toxicological effect of engineered nanomaterials on the liver. Br. J. Pharmacol., 171, 3980–3987. 24111818 10.1111/bph.12421 PMC4243972

[63] de Oliveira Mallia J. Singh N. Bohic S. Shah U. K. Jenkins G. J. and Doak S. H (2016) Assessing genotoxicity of iron oxide nanoparticles within an in vitro liver 3D model. Toxicologist., 150(1), Abstract no. 2772.

[64] Maurici D. Aardema M. Corvi R. (2005) Genotoxicty and mutagenicity. Altern. Lab. Anim., 1, 117–130. 10.1177/026119290503301s13 16194145

[65] Mun G. C. Aardema M. J. Hu T. Barnett B. Kaluzhny Y. Klausner M. Karetsky V. Dahl E. L. and Curren R. D (2009) Further development of the EpiDerm 3D reconstructed human skin micronucleus (RSMN) assay. Mutat. Res., 673, 92–99. 19167515 10.1016/j.mrgentox.2008.12.004

[66] Park E. -J. Umh H. Kim S. -W. Cho M. -H. Kim J. -H. and Kim Y (2014) ERK pathway is activated in bare-FeNPs-induced autophagy. Arch. Toxicol., 88, 323–336. 24068039 10.1007/s00204-013-1134-1

[67] Park B. Martin P. Harris C. Guest R. Whittingham A. Jenkinson P. and Handley J (2007) Initial in vitro screening approach to investigate the potential health and environmental hazards of Enviroxtrade mark: a nanoparticulate cerium oxide diesel fuel additive. Part. Fibre Toxicol., 4, 12. 18053256 10.1186/1743-8977-4-12 PMC2245973

[68] Choi J. Kim H. Choi J. Oh S. M. Park J. and Park K (2014) Skin corrosion and irritation test of sunscreen nanoparticles using reconstructed 3D human skin model. Environ. Health Toxicol., 29, e2014004. 25116366 10.5620/eht.2014.29.e2014004 PMC4152941

[69] Hu T. Kaluzhny Y. Mun G. C. Barnett B. Karetsky V. Wilt N. Klausner M. Curren R. D. and Aardema M. J (2009) Intralaboratory and interlaboratory evaluation of the EpiDerm 3D human reconstructed skin micronucleus (RSMN) assay. Mutat. Res., 673, 100–108. 19167513 10.1016/j.mrgentox.2008.12.003

[70] Aardema M. J. Barnett B. C. Khambatta Z. (2010) International prevalidation studies of the EpiDerm 3D human reconstructed skin micronucleus (RSMN) assay: transferability and reproducibility. Mutat. Res., 701, 123–131. 20621637 10.1016/j.mrgentox.2010.05.017

[71] Roy S. Kulkarni R. Hewitt N. J. and Aardema M. J (2016) The EpiDerm™ 3D human reconstructed skin micronucleus (RSMN) assay: Historical control data and proof of principle studies for mechanistic assay adaptations. Mutat. Res., 805, 25–37. 10.1016/j.mrgentox.2016.05.010 27402480

[72] Chapman K. E. Thomas A. D. Wills J. W. Pfuhler S. Doak S. H. and Jenkins G. J (2014) Automation and validation of micronucleus detection in the 3D EpiDerm human reconstructed skin assay and correlation with 2D dose responses. Mutagenesis, 29, 165–175. 24675152 10.1093/mutage/geu011 PMC3983754

[73] Wills J. W. Hondow N. Thomas A. D. (2016) Genetic toxicity assessment of engineered nanoparticles using a 3D in vitro skin model (EpiDerm). Part. Fibre Toxicol., 13, 50. 27613375 10.1186/s12989-016-0161-5 PMC5016964

